# Modeling of Gate Stack Patterning for Advanced Technology Nodes: A Review

**DOI:** 10.3390/mi9120631

**Published:** 2018-11-29

**Authors:** Xaver Klemenschits, Siegfried Selberherr, Lado Filipovic

**Affiliations:** Institute for Microelectronics, Technische Universität Wien, Vienna 1040, Austria; selberherr@iue.tuwien.ac.at (S.S.); filipovic@iue.tuwien.ac.at (L.F.)

**Keywords:** technology computer-aided design (TCAD), metal oxide semiconductor field effect transistor (MOSFET), topography simulation, metal gate stack, level set, high-k, fin field effect transistor (FinFET)

## Abstract

Semiconductor device dimensions have been decreasing steadily over the past several decades, generating the need to overcome fundamental limitations of both the materials they are made of and the fabrication techniques used to build them. Modern metal gates are no longer a simple polysilicon layer, but rather consist of a stack of several different materials, often requiring multiple processing steps each, to obtain the characteristics needed for stable operation. In order to better understand the underlying mechanics and predict the potential of new methods and materials, technology computer aided design has become increasingly important. This review will discuss the fundamental methods, used to describe expected topology changes, and their respective benefits and limitations. In particular, common techniques used for effective modeling of the transport of molecular entities using numerical particle ray tracing in the feature scale region will be reviewed, taking into account the limitations they impose on chemical modeling. The modeling of surface chemistries and recent advances therein, which have enabled the identification of dominant etch mechanisms and the development of sophisticated chemical models, is further presented. Finally, recent advances in the modeling of gate stack pattering using advanced geometries in the feature scale are discussed, taking note of the underlying methods and their limitations, which still need to be overcome and are actively investigated.

## 1. Introduction

Ongoing miniaturization of metal oxide semiconductor field effect transistors (MOSFETs) is essential for the continued advances in computing performance, reduction of chip area, and lowered power dissipation in modern integrated circuits in accordance with Moore’s Law [1]. For decades, the design of MOSFETs did not change drastically [2], while its size was scaled thanks to more advanced lithography techniques and improved fabrication processes. Additionally, thinner insulating layers and smaller dimensions allowed for faster switching, thereby increasing speed and improving performance. However, smaller sizes presented new challenges. For example, the insulating silicon dioxide (SiO_2_) layer between the conducting channel and the gate became so thin that quantum tunneling resulted in gate leakage currents too high to sustain stable MOSFET operation [3]. However, the insulating SiO_2_ layer is required to be as thin as possible in order to reach the high gate to channel capacitance required for effective switching characteristics, while a physically thicker layer helps to reduce tunneling. Effective switching and reduced tunneling were achieved by replacing SiO_2_ with a material with a higher dielectric constant (high-k material), to balance the increased distance between the gate and the channel. The most prominent of those materials used today are Hafnium (Hf)-based insulators, usually HfO_2_. The combination of a high dielectric constant and a wide band gap [4], needed to create a potential barrier to the silicon channel and thus to act as an insulator, make HfO_2_ ideal for this purpose. Therefore, it is the most commonly used material for gate insulation ever since its introduction in the 45 nm technology node [5]. However, new materials, such as Al_2_O_3_, are currently being investigated as possible alternatives [6].

The gate contact material must be chosen carefully, since its work function controls the threshold voltage, above which the channel will be inverted. Fine control over this important parameter of a MOSFET was achieved with a polycrystalline silicon (Poly-Si) gate, doped depending on the type of transistor desired. The dopant concentration inside the gate material influences the work function and thus allows the channel band to be shifted either towards or away from the Fermi energy level, decreasing or increasing the threshold voltage, respectively. However, doping of the gate contact results in the unwanted penetration of dopants into the dielectric and the channel, leading to numerous unwanted side effects [7]. Furthermore, other unfavorable characteristics of Poly-Si, such as Fermi pinning and gate depletion [8], created the need for different materials to be considered for the gate contact and led to the re-introduction of metals into the gate stack. However, not aluminum, but rather titanium nitride (TiN) is nowadays the most commonly used material, since its work function is close to the middle of the silicon band gap, meaning that it can be used for both p-type and n-type transistors and does not require doping [9]. An additional benefit of TiN over Poly-Si is that it has a lower electrical resistance and can act as an oxygen diffusion barrier, increasing the stability of the dielectric. Furthermore, its integration into fabrication is simple since it has already been used in the complementary metal oxide semiconductor (CMOS) fabrication process as a dielectric in interconnects and for diffusion barriers. However, the threshold voltage in a metal gate stack can only be tuned by doping the channel itself, degrading some of its characteristics. Therefore, there has been a considerable interest in finding advanced gate metals of desirable work functions [10]. Among others, TiC [11], TiAlC [12], and Ru [13] are heavily investigated as potential future materials.

The introduction of new materials inevitably led to the need for more complex fabrication techniques and intricate patterning steps to achieve smaller feature sizes as laid out in the International Roadmap to Semiconductors (ITRS) [14] and the International Roadmap for Devices and Systems (IRDS) [15]. Several complex deposition, etch, and cleaning steps are necessary in order to manufacture highly controlled gate profiles without damaging neighboring materials. The introduction of three-dimensional structures such as fin field-effect transistors (FinFETs) has added further complexities to the gate patterning process [16], as straight etch profiles must be obtained despite the exposure of sections of the underlying material. Therefore, a combination of highly directional as well as selective patterning techniques and intermediate cleaning steps must be applied in order to achieve the required accuracies [6].

In order to develop new techniques for reliable patterning of more and more complex gate structures and to improve the understanding of the underlying mechanisms, modeling becomes increasingly important. On one hand, a comparison of simulation results with experimental data can give insight into the physical properties of different processes, as disagreements between the two indicates the presence of additional phenomena, which must be considered. On the other hand, reliable and predictive models for processing steps allow for quick testing of new designs without the need for expensive experiments. Especially in the case of gate stack etching sequences, such predictive models can reduce development costs greatly as the fabrication of prototypes is expensive and time-consuming. However, complex gate structures require the careful combination of the different etch techniques described above, including their influence on the subsequent etch steps, resulting in the need for sophisticated modeling of the underlying physical phenomena. In order to enable such complex simulations, even fundamental computational techniques must be considered carefully to achieve physically meaningful results. Therefore, this review will cover the fundamental techniques of process simulation, such as methods for describing moving surfaces and particle transport inside a plasma chamber, and move on to modern patterning techniques and sophisticated models used to describe them. The particular aim of this review is to summarize recent achievements in the simulation of the etching of advanced node multi-layered gate stacks.

## 2. Methods

The underlying numerical methods driving simulators have important implications on the modeling capabilities. It may be highly inefficient or even impossible to model certain physical processes using a specific method, but straightforward using another. Therefore, the choice of appropriate numerical methods depends strongly on the desired modeling capabilities. In order to judge the applicability of a certain method to a problem, a deep understanding of the relevant physics, as well as of the method itself is necessary. The fundamental methods used to describe the wafer surface and the flow of atoms, ions, and molecules within the feature scale are discussed in this section, highlighting their respective consequences to the final modeling capabilities of a simulator.

### 2.1. Surface Representations

The simulation of microelectronic fabrication techniques requires accurate descriptions of the topography of different materials and their interfaces, since certain processing steps, such as deposition and etching, can result in complex surface deformations. Therefore, the ability of the surface description to represent such changes over time in a robust way is essential. The manner in which the surface will move is usually calculated for every surface element and applied for a discrete time step [17], to be calculated again for the resulting new topography [18]. The manner in which this surface evolution is applied depends strongly on the surface representation used. The surface representation can be explicit or implicit, both of which are addressed in this section. Implicit surface descriptions have become standard in modern technology computer-aided design (TCAD) simulators due to their robustness and computational efficiency.

#### 2.1.1. Explicit Surfaces

Many applications, such as graphics rendering, rely on explicit surface representations which define surface elements by interconnected points on the surface [19]. This representation has several desirable properties, such as no principal limitation on feature size or resolution and minimal memory requirements, since the number of surface elements scales directly with the total area [20]. Furthermore, it can be visualized easily, since the absolute coordinates of all elements are known by design. Therefore, all volume and surface elements have fixed sizes, which is useful when modeling stress, which can develop when an element grows in size while surrounded by other elements. One such process is oxidation, where the substrate below a mask grows and moves the mask, leading to stress within the materials [21].

**Figure 1:**
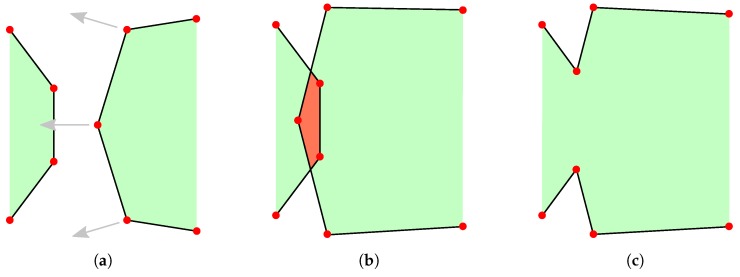
Two explicit surfaces merging by movement of nodes: (**a**) initial geometry with included area (green); (**b**) broken final geometry due to a surface overlap (red); (**c**) correct surface after merging of the two surfaces. In order to reach the correct surface representation, additional meshing steps are necessary, decreasing performance.

The movement of a surface is realized by shifting the defined nodes in a desired direction and connecting the points again to obtain new surface elements [22]. This can lead to a non-physical intersection of surface elements as shown in Figure 1, since there is no strict definition of which side of the surface represents the material and which one is the ambient space [19]. Figure 1 shows the merger of two surfaces, which creates a non-physical geometry in the center, due to the overlap of two materials. This is a common concern with explicit surface definitions. Testing for such self-intersections is a computationally expensive process and thus not favorable when describing moving surfaces. The separation of surfaces, or indeed any movement of a surface, can lead to similar problems as different nodes must be identified and connected correctly to achieve accurate descriptions of the interfaces [23]. Furthermore, topography changes can lead to a wide separation of neighboring points and therefore large surface element areas, reducing the accuracy of the surface representation. Due to these potential problems, the surface must be remeshed regularly to obtain proper and efficient representations after each time step. This includes recalculating nodes and surface elements in order to satisfy certain minimum mesh quality criteria, such as equal area triangles or equal edge lengths [24]. This additional step can be computationally expensive for large surfaces and is therefore not desirable in complex, three-dimensional simulations.

#### 2.1.2. Implicit Surfaces

Implicit surfaces are isosurfaces described by a function $\phi (\vec{x})$, defined at every point in space. It is not solved for one of its variables, but rather used to find the set of points which let the function go to a specific scalar value, usually zero [23,25]. Therefore, all points on the surface, $\vec{x}\in S$, must satisfy $\phi (\vec{x})=0$, which is why these points are called the zero level set. Since it is not feasible to represent all possible surfaces algebraically, $\phi (\vec{x})$ is constructed using signed distance transforms. These construct $\phi (\vec{x})$ from the distance *d* between any point $\vec{x}$ and the surface *S* bounding the volume *M*:

(1) $$\phi \left(\vec{x}\right)=\left\{\begin{array}{cc} -d, & \mathrm{for}\;\vec{x}\in M,\\ 0, & \mathrm{for}\;\vec{x}\in S,\\ d, & \mathrm{for}\;\vec{x}\notin M. \end{array}\right.$$

Therefore, every point in the simulation domain is known to be inside or outside *M*, by examining the sign of $\phi (\vec{x})$, without the need for further analysis. Robust and fast algorithms for signed distance transforms exist [26,27,28], allowing for simple integration into process simulators. These algorithms construct $\phi (\vec{x})$ from an explicit representation, such as a triangulated surface, by traversing the simulation domain and finding the smallest distance between $\vec{x}$ and the surface iteratively. The fast marching method is optimized for level sets and thus it is the most efficient method in converting between explicit surfaces and level sets [29].

The time evolution of a surface is usually captured in a scalar field denoting the surface normal speed $v(\vec{x})$ [30]. For simple surfaces, such as planes, the velocity field can be subtracted from the signed distance function to move the surface with velocity $v(\vec{x})$:

(2) $$\frac{\partial \phi (\vec{x},t)}{\partial t}=-v\left(\vec{x}\right).$$

However, more complex surfaces with non constant gradients must be moved differently in order to retain their shape, which is achieved using the gradient of the signed distance function to normalize $v(\vec{x})$, leading to the level set equation [31]:

(3) $$\frac{\partial \phi (\vec{x},t)}{\partial t}+v\left(\vec{x}\right)\left|\nabla \phi \left(\vec{x},t\right)\right|=0.$$

**Figure 2:**
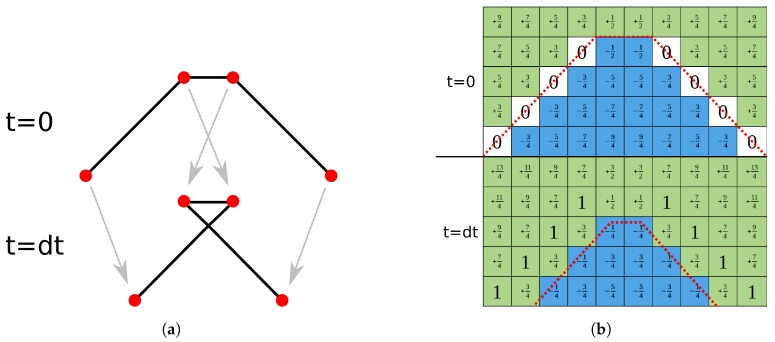
Schematic comparison between explicit and implicit surfaces being moved, highlighting self-intersection in explicit surfaces. In contrast, the level set method applied in (**b**) shows surface movement without self-intersection, albeit losing some features such as the expected sharp peak in the center of the geometry. (**a**) Nodes of an explicit surface moved by a velocity field, creating a self-intersection. Additional steps are required to form a correct representation of the surface. (**b**) Implicit surface being moved by adding unity to all values stored on a regular grid. The numbers represent the level set value stored at the center of each cell. Negative regions inside the surface are highlighted in blue, regions outside with positive values in green. Dashed, red lines indicate the explicit location of the surface.

Since Equation (3) is a form of the Hamilton–Jacobi equation, often encountered in mathematics, many algorithms are available to solve it using finite difference schemes [31,32]. In order to use $\phi (\vec{x})$ in numerical simulations, the values of the function are usually stored at points defined on a regular grid to achieve an approximate representation, as shown in Figure 2b. The regular spacing of grid points enables the use of well-known finite difference algorithms to solve the differential equations needed for the calculation of surface normal vectors, surface curvature, or the time evolution as described above [33].

Since it is not the exact location of the surface, which is stored but the distance to it at regular intervals in the entire simulation domain, the position of the grid points does not change with the moving surface, but only their respective level set values. This means that self-intersection and similar problems occurring in explicit surfaces are not encountered using implicit level set methods. Figure 2 shows these clear differences by comparing the movement of a surface in these representations. Figure 2b also highlights another characteristic of the level set method, which is the loss of sharp features on the surface [34]. As can be seen clearly, the peak expected after the evolution of the surface is flattened due to the size and limited resolution of the grid. Increasing the number of grid points will dampen this effect, however, increasing the computational cost greatly, since the number of grid points scales with the domain volume. Nevertheless, these negative effects are not expected to reduce the quality significantly because the modeled processes do not tend to create radically outstanding features, but rather smooth profiles varying over a number of grid points. Nevertheless, great care must be taken when choosing the number of grid points to balance computational cost with simulation accuracy. Additionally, the spacing of grid points influences the output of numerical schemes for calculating the curvatures or normal vectors at grid points. Therefore, the accuracy is influenced by the chosen grid resolution in several ways.

**Figure 3:**
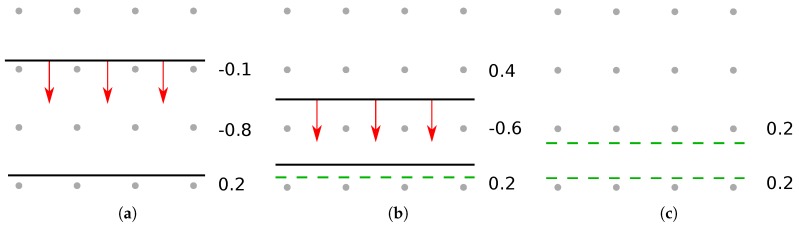
Top surface of a thin layer is moved until the layer disappears entirely. Grey points indicate the grid, black lines the surface, red arrows the movement per time step, and dashed green lines the correct position of the surface. The level set values of each row are shown to its right. (**a**) Initial layer, only two grid spacings wide, whose top surface is moved downwards. (**b**) As the layer is thinned to only one grid point, the level set values are not normalized anymore, resulting in symmetric shrinking. (**c**) Once the last row of grid points is outside of the layer, it ceases to exist, although it should still be almost one grid spacing wide.

Furthermore, the merger or separation of surfaces does not require additional consideration as there is no ambiguity about whether a point lies inside or outside the surface. Hence, two surfaces growing towards each other must merge, when there are no more oppositely signed points between them, since there can be no part of the encapsulated volume between them. However, this can lead to surfaces merging too quickly, when there are no oppositely signed points between the fronts, leading the surface evolution to jump up to one grid spacing just before merging. The same effect can be observed for separating surfaces or thin layers being removed entirely, which is shown in Figure 3. Therefore, the grid spacing also sets a minimum layer thickness in all directions.

**Figure 4:**
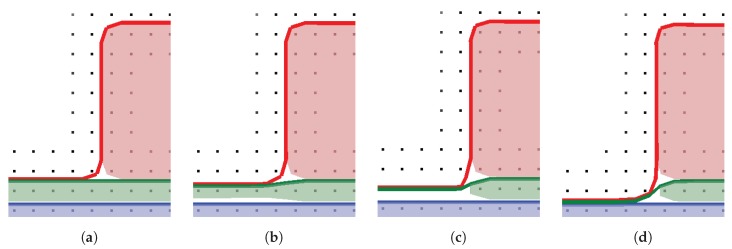
Schematic comparison of the difference in surface representations when layers are wrapped around lower ones (volume inside solid lines) or if they only encapsulate a single material (colored areas) in uniform etching of a thin layer (green) under a mask (red). Different problems when representing only a thin layer with level sets are shown. (**a**) Initial layout with only minor discrepancies between the two representations. (**b**) Symmetric shrinking of a single layer as the level set value in the center decreases: the bottom of the thin layer (green) lifts up as there is only one grid point defining the distance, reducing the layer symmetrically as shown in Figure 3b. (**c**) Complete removal of the thin layer as no grid point is inside the surface anymore. (**d**) Final layout with receded surfaces: the thin layer is still intact for wrapped level sets, but is completely removed for individual materials. (used from [35] under CC BY 4.0 / cropped from original).

Especially in modern gate stack etching sequences, the accurate description of thin layers is of critical importance to the combination of different chemical processes, which deposit thin layers of different materials while etching the structure. These thin layers have a considerable impact on the subsequent etch steps as they have very different chemical properties to the substrate. Despite being very thin, they can protect the underlying material from etching, since they might etch very slowly. If, however, they are ignored or disappear too early in the simulation, the underlying material is exposed, resulting in an inaccurate modeling of the physical process. The problem of quick merging and the disappearance of layers, as well as the symmetric shrinking is usually due to few grid points and therefore a lack of information about the surface position. This can be overcome by describing thin layers by not only the material they consist of, but also including the sum of all materials beneath them [35]. The materials below are also stored as a separate level set so that the original thin layer is not lost and can be extracted again. The advantages of this material representation over defining single materials separately is highlighted in Figure 4, which strongly increases accuracy, especially when considering the thin layers. In order to recover the single materials, the lower ones must be subtracted from the top material, which can be performed efficiently using the level set method, since any boolean operation can be carried out element-wise at each grid point [36]. The intersection between two level sets, for example, can be achieved by comparing the two values at each grid point and choosing the greater of the two. This results in a stable conversion to single layers, although thin materials might not be represented correctly in a separate level set, due to the effects described above.

The memory requirements for storing a level set surface are high compared to explicit surfaces, as they scale with volume rather than with surface area [31]. Figure 2b highlights that only the points around the surface are needed to describe the set of zeros defining it, as the surrounding values increase linearly in an ideal level set. Therefore, only a few layers around the boundary, a so-called narrow band [37], influence the surface description. If the surface evolves towards the edge of the narrow band, a new band must be initialized with the surface at its center. Since re-initialization is computationally expensive, there is an optimal width of the narrow band, which uses the smallest number of grid points for calculations and avoids re-initialization for as long as possible. The optimal width found in the original publication [37], was between 6 and 12 layers of active grid points. An extension of the narrow band approach is the sparse field algorithm, which significantly reduces computational cost of re-initialization by approximating distances from the surface in a stable way [33]. Using the sparse field algorithm, re-initialization can be performed at every time step, which allows for the use of only a single layer of grid points, thus achieving optimal storage efficiency [38]. Neighboring grid points for the calculation of surface normals and curvatures can be calculated for each time step using the same efficient distancing algorithm.

#### 2.1.3. Cell Based Methods

Another common approach to describing surfaces is considering them only as interfaces between different materials. By describing only the volume occupied by a material, the interfaces are simply described by its boundaries. In cell-based methods, this is usually realized on a regular grid, where every grid point represents a unit cube, or voxel, at its location, storing relevant information [39]. Usually, a number denoting the material and a filling fraction are stored, allowing for the calculation of the exact location of the boundary [40].

At high resolutions, it is also possible to describe geometries using only a single material per cell, which allows for simpler modeling. If no filling fraction is stored, but only the material of the cell (i.e., the filling fraction is binary), this method can also be considered a voxel based explicit surface representation [41]. The geometry can be extracted easily, as each voxel can be included explicitly, although this leads to stepped surfaces. If the cell size is close to the size of physical atoms, cell based methods can come close to atomistic modeling, enabling more accurate descriptions of physical processes, such as diffusion or ion implantation [42].

**Figure 5:**
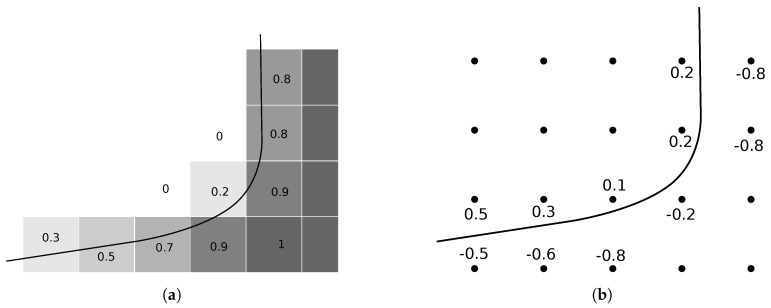
Comparison of cell-based and level set representations in narrow-band implementations. While the former is intrinsically associated with volume, level set representations describe an interface or boundary. (**a**) Cell-based representation of an explicit surface (black line) with numbers indicating the filling fraction of each cell. The darker a cell, the higher the filling fraction. (**b**) Level set representation of an explicit surface (black line) with level set values, related to the normal distance from the surface, for each grid point.

As shown in Figure 5, this approach is similar to the level set method because it represents the boundaries implicitly on a regular grid. However, this leads to the same shortcomings, such as resolution limitations and scaling problems. These can be overcome with similar techniques as described above, such as the narrow band approach. Cell-based methods share their robustness for complex topographies with other implicit methods. Moving a cell based surface is more trivial than one defined by level sets, as conservation of mass can be used to add or remove volume from a voxel, greatly simplifying surface velocity calculations.

However, numerical schemes associated with cell based methods are not as efficient as those used in level set representations [43] and if filling fractions are non-binary, conversion to explicit surfaces is complex [44]. Since there is no information about where the material lies inside a cell, the explicit boundary must be reconstructed from the surrounding cells. This can be quite complex, especially when considering several different materials or thin layers of materials, where there is little material within a cell while it stretches across the entire cell width. Reconstructing an explicit surface might thus be ambiguous and not reliable for complex structures. Therefore, most simulating frameworks use level set representations for moving surfaces as they usually are more robust to complex deformations and computationally more efficient when modeling large structures. Cell based methods are better suited for describing smaller geometries, incorporating mixed materials and volume characteristics, such as implanted ions.

### 2.2. Surface Velocity Calculation

As outlined in Section 2.1, the surface evolution is governed by a scalar field of velocities $v(\vec{x})$, describing how much each discretized element of the surface should move in the next time step. Therefore, the most crucial part in process simulation is the calculation of those velocities using models, which match the described physical process as closely as possible, whether empirically or physically. Despite recent advances in atomistic modeling [45], the structures considered in process simulations are usually too large to take into account individual atoms and, therefore, the surface is approximated as a continuum, using the surface representations described in Section 2.1. This results in the loss of microscopic information, such as surface roughness, while considerably decreasing computational complexity [46]. Velocities must be calculated for every discretized surface element, in order to move the surface correctly. Therefore, a velocity value for each triangle of an explicit surface, for each grid point of a level set, and for each cell of a cell-based representation must be defined in order to advance the surface.

A simple way to generate these velocities, is to extract geometric parameters, such as etch depth, from experimental data and changing the surface to replicate the result of the fabrication process. This approach is called process emulation, as no physical behavior is modeled, but rather simple geometric rules are applied to mimic the result of a fabrication process [47]. Constant deposition, for example, can be approximated by expanding the surface by the same amount in each direction, meaning the growth rate is the same everywhere on the surface. More complex processes can also be emulated by applying more sophisticated geometric rules [48]. Since no physical processes have to be modeled, this approach is computationally efficient. Therefore, this method is useful for creating large structures quickly for device characterization or for feasibility studies, due to its high efficiency [49,50,51]. However, it is not very accurate, especially when describing complex processing steps.

Since process emulation does not take into account any physical properties of the surface or the etch chemistry, it cannot be used for any physical analysis. In order to identify dominant etch or deposition mechanics, or even predict the properties of new fabrication processes, a sophisticated physical description of the involved physics and chemical reactions is necessary. This approach is called process simulation and is focused on in the following sections, which cover the modeling of the transport of atoms, ions, and molecules through the feature scale region, as well as the modeling of surface reactions leading to etching or deposition. From these models, the surface velocity field $v(\vec{x})$ can be generated, leading to a physically accurate deformation of the simulated wafer surface.

### 2.3. Transport of Molecular Entities in Plasma Environments

**Figure 6:**
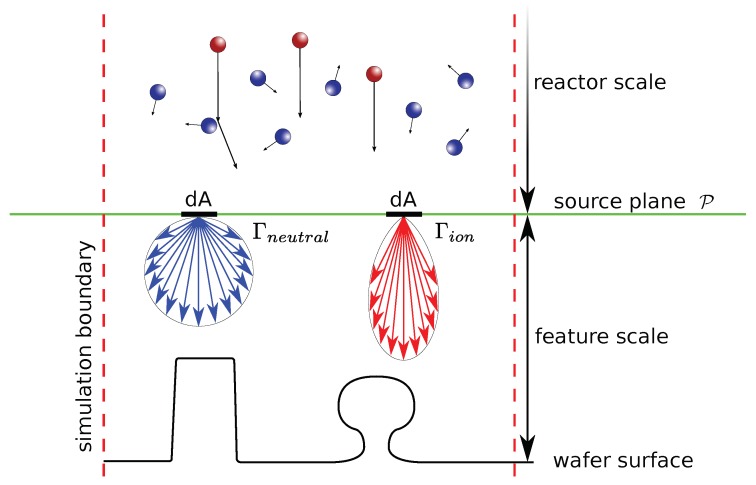
Schematic representation of the traversal of neutral molecular entities and ions through the reactor and feature scale regions. While the motion through the reactor scale is dominated by random collisions with other molecular entities, the path through the feature scale region is dominated by ballistic transport. The directional distribution of neutral atoms and molecules $\Gamma_{neutral}$ (blue) and ions $\Gamma_{ion}$ (red) entering the feature scale region is shown as blue and red arrows, respectively. The molecular entities will then traverse the feature scale region in straight lines, only colliding with the surface.

In order to simulate how much material is removed or deposited on a surface during a fabrication process, the rates at which different atoms, ions or molecules impinge on the surface must be found. Collectively, atoms, ions and molecules are hereafter referred to as molecular entities. These rates can depend strongly on different geometrical effects and transport phenomena inside the reactor [52]. The way in which molecular entities traverse the reactor depends on their specific properties, as well as on the thermodynamics of the chosen process. In order to describe this transport, the reactor space is usually divided into reactor-scale and feature-scale regions separated by a plane P, as shown in Figure 6. This simplifies the description of neutral atoms and molecules because their motion in the reactor-scale region is governed by the Maxwell–Boltzmann distribution, since this region is large compared to their mean free path [53]. In contrast, the feature-scale region is small in relation to their mean free path, meaning collisions with the surface are much more common than those with other parts of the gas phase. Therefore, ballistic transport is commonly used to describe the propagation of molecular entities through the feature scale region [54], which can result in shadowing and reflection effects. This transport can then be simulated in a straightforward manner using ray tracing methods. Knudsen diffusion has also been used successfully to describe the transport of atoms, ions and molecules in simple geometries, such as straight trenches [55,56], eliminating the need for complex modeling of the molecular entities’ trajectories. However, a process description close to the physical reality can only be obtained by considering particle transport directly.

Physically, each infinitesimal element dA on P can be considered as an individual source of molecular entities with certain properties and emitting fluxes, as highlighted in Figure 6. Usually, the angular dependence of the neutral molecular entities’ distribution $\Gamma_{neutral}$ is assumed to follow a cosine due to the angular projection of dA on the emission angle with P [57]. The distribution of accelerated ions $\Gamma_{ion}$, present in ion-enhanced plasma etching processes, are usually described by more focused power cosine or normal distributions [46]. This leads to highly directional properties which reflect those found in experiments. In order to describe the motion of ions as straight trajectories in the feature scale region, the electromagnetic field distortion by the surface must be small enough to not influence the paths of ions drastically, which is a reasonable assumption given the short path lengths of ions in the feature scale region and the strong directional electric fields used to guide the ions.

When the physics described above are simulated, the reactor and feature scale are usually treated separately, as the only input needed for feature scale simulations are the source distributions $\Gamma_{neutral}$ and $\Gamma_{ion}$. These can be obtained from experiments or from chemical kinetic simulations. Simulating the feature scale requires some additional consideration, due to the limited size of the simulation domain and other computational limits. For example, due to the large number of molecular entities in a physical process, it is not feasible to simulate all of them. They are usually simulated with Monte Carlo techniques using particles, where each particle represents multiple molecular entities. Computational methods addressing this problem are described in the following sections.

Another important factor for simulations is the appropriate choice of boundary conditions due to the limited size of the simulation domain, when compared to the size of an actual wafer. If the simulated wafer contains only a single structure which fills the simulation domain and is planar otherwise, particles which leave the simulation domain can be ignored, since they cannot return back into it. However, if the same structure is repeated across the wafer, periodic boundary conditions are more appropriate since particles which are reflected to leave the domain can be mapped back into it, as if they originated from the neighboring structure, increasing simulation accuracy. This enables the consideration of parts of the wafer, which cannot be simulated directly due to the limited size of the simulation domain. The same applies to reflective boundary conditions, where the neighboring structures are mirrored to the considered one.

#### 2.3.1. Top-Down Flux Calculation

The fluxes at which different particles impinge on each part of the surface can be found by launching a large number of particles from the source plane P and using ray tracing to find the point of impact of each particle on the surface [58]. After all particles have been traced, the number of impacts is counted for each discretized surface element, which may differ depending on the surface representation used. Each simulation particle may represent a single molecular entity or several of the same species, depending on the number of particles used to simulate the transport. Simulating the maximum number of particles, each describes only a single molecular entity. However, this is not practical due to the large number of actual molecular entities usually involved in a physical fabrication process. Therefore, fewer particles are used, each representing a number of molecular entities. The particle flux at each discretized surface element is then found by the number of incident particles times the number of molecular entities represented per particle.

**Figure 7:**
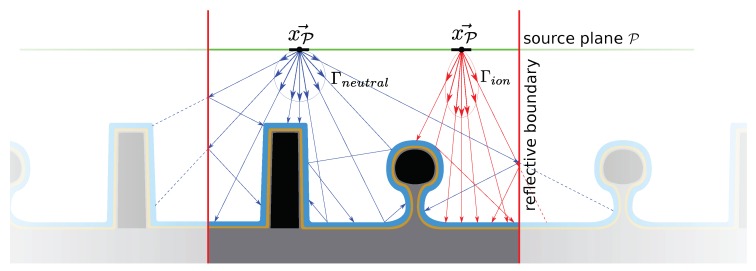
Schematic depiction of discrete particles being traced from the source plane to the surface using rays. Each particle either describes neutral atoms or molecules (blue) or ions (red), governed by the source distributions $\Gamma_{neutral}$ and $\Gamma_{ion}$, respectively. They define the relative probability of particle direction, energy and other properties. Specular reflections are shown for ions, and diffuse reflections for neutral species.

Monte Carlo methods are employed to generate particles according to the probability distributions describing the neutral flux $\Gamma_{neutral}$ and ion flux $\Gamma_{ion}$ [59], while ray tracing methods, as used in computer graphics [60], enable the simulation of a large number of particles. These are launched from several points on the source plane P, usually spaced at regular intervals, forming a grid of particle sources. A large enough quantity of particles thus results in a good description of the effect on the surface of the original source distribution. Figure 7 schematically shows how particles, launched from different locations of the source plane in pseudo-random directions, might interact with the surface, with some particles experiencing multiple reflections. The starting direction and the probability of reflection and re-emission are both determined probabilistically, meaning numerous pseudo-random numbers must be generated, increasing the performance requirements [61].

Balancing computational efficiency with simulation accuracy is one of the main concerns in this method, as the modeling complexity can theoretically be extended to model every single molecular entity without systematic limits, due to the physical nature of this approach. However, the computational cost of tracing a large number of particles is high as several intersection tests with the surface have to be performed for each particle [46]. The minimum number of particles depends on the exact implementation and whether smoothing is used in order to avoid abrupt changes in the fluxes along the surface. In general, it must be ensured that each discretized surface element is intersected several times, if one is to achieve physically meaningful results. This means that several particles must reach each triangle in explicit surfaces, grid point in level sets, or cells in cell-based methods. The complex geometries used in modern gate stacks require a large number of particles in order to create a physically meaningful flux profile everywhere on the surface.

**Figure 8:**
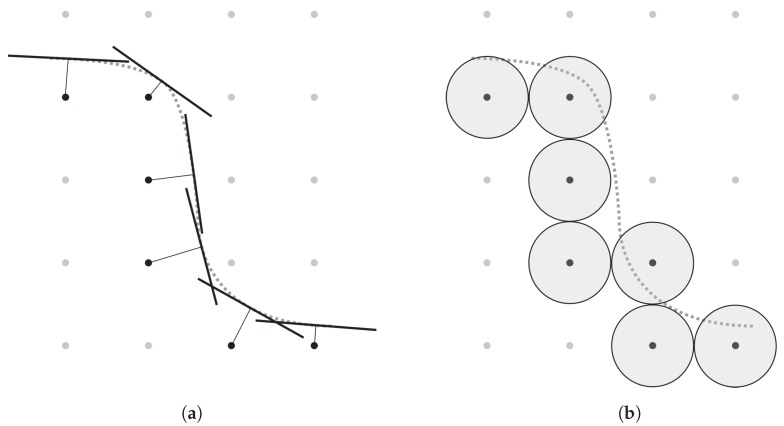
Two ways to approximate an implicit surface efficiently by explicit shapes on active grid points (black), in order to simplify intersection tests for ray tracing. The line segments and circles are replaced by disks and spheres in three dimensions. (**a**) tangential line segments used to form an explicit approximation of the surface, as described in [57]; (**b**) surface approximated by explicit circles centered at active grid points, as described in [63].

Although implicit surface representations are often encountered in process simulations, as well as visualization tasks, some form of explicit surface is usually required in an intermediate step during ray tracing. An explicit surface representation is often necessary, since intersection tests used in ray tracing algorithms are much more efficient on explicit surfaces than on implicit ones. Once the fluxes have been found and the velocity field $v(\vec{x})$ is generated, an implicit representation can be used again to move the surface. Conversion between the two representations can be quite time-consuming, creating a bottle-neck for simulation efficiency. However, perfectly closed explicit surfaces are not strictly required for ray tracing as small self-intersects and other minor flaws in the geometry do not have a great effect on the final result. Intermediate, explicit surfaces can be created by triangulated, cell-based approximations of the surface as produced by marching cubes algorithms [62] or more crudely by approximating the surface using discs [57] or spheres [63]. In this approach, each implicit grid point is approximated explicitly by a disc or sphere with a radius of at least one grid point separation, resulting in a closed surface due to the overlap of discs or spheres, as shown in Figure 8. Spheres can be placed directly on the grid points and do not require any translation to the surface normal, making them more efficient, albeit less accurate. This allows for quick conversion between the surface representations, while still enabling the use of advantageous explicit ray tracing methods.

Due to the physical nature of the top-down method, even complex reflective properties, such as specular reflection, can be modeled straightforwardly, as the incoming angle of a ray is found easily from the intersection test and extracting the surface normal and curvature is intuitive when using the level set method [25]. If diffuse reflections are to be considered, some form of random number generation must also be applied several times per ray to find the reflected direction, which increases the simulation time. Additionally, other effects, such as different material properties due to variations in the crystal orientation [64], can be included for a more physical description. This approach also allows for particle–particle collisions to be considered, if the simulated geometries are too large for the assumption of ballistic transport to hold. This can be the case for large aspect ratio geometries, where particles may travel far in one direction without a surface intersection. Therefore, due to the physical approach of this method, it is the most accurate one as it does not limit the number of effects which can be included in modeling the physical processes. However, it usually requires more computational effort than alternatives.

#### 2.3.2. Bottom-Up Flux Calculation

**Figure 9:**
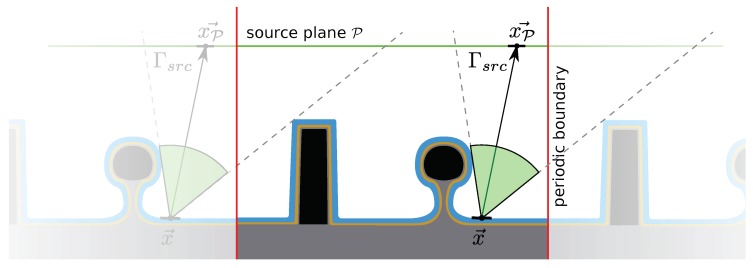
Schematic representation of the bottom-up flux calculation for modern gate structures, using periodic boundary conditions, meaning the entire simulation domain is repeated at the boundaries. The black arrow indicates the direction used to find the direct flux incident on $\vec{x}$ from a single particle source at $\vec{x_{P}}$ with a source distribution of $\Gamma_{src}$. The flux of all visible source plane elements, indicated by the green arc, is summed to give the total direct flux on $\vec{x}$.

As mentioned in Section 2.3.1, the source plane P is usually described numerically as a regular grid of particle sources. Instead of tracing many rays from each source to the wafer surface, it is also possible to do the reverse. In the bottom-up method, a single discretized surface element is considered, and all the particle sources visible to it are summed [65,66]. This is achieved by iterating over all discretized particle sources on the source plane P. For each source, it is verified whether the source, located at $\vec{x_{P}}$, is visible from the considered discretized surface element at $\vec{x}$ (Figure 9). The particle flux incident on this point on the surface is then found by considering the particle source distribution $\Gamma_{src}$. Summing the contributions of all particle sources gives the total particle flux incident on the discretized surface element at $\vec{x}$:

(4) $$F_{0}\left(\vec{x}\right)=\sum_{\vec{x_{P}}}\Gamma_{src}\left({\vec{x}}_{P},\vec{x}\right)Y\left(\vec{x_{P}},\vec{x}\right).$$

Here, the angular dependence of the source is captured by $\vec{x_{P}}$ and $\vec{x}$, as their relative positions give the relevant angle of emission and impact, respectively. The visibility function, $Y(\vec{x_{P}},\vec{x})$, describes whether a particle source at $\vec{x_{P}}$ is visible to a surface element at $\vec{x}$, and is unity if the point is visible and zero otherwise. Visible points are indicated by a green arc in Figure 9, so the fluxes of all discretized particle sources within this arc are included in the total flux.

Equation (4) only gives the particle flux incident on the surface directly from the source plane and does not include any reflection or re-emission effects from other locations on the surface. It is possible to formulate an analytical solution to include reflection and re-emission and numerically solve for the total flux [66]. However, this approach can be memory intensive for large geometries due to the large matrices built to describe the correlations between large numbers of discretized surface elements and particle sources on the source plane [67]. For highly symmetric geometries, such as high aspect ratio holes or trenches, the calculations can be simplified by considering their symmetries and calculating the fluxes only for non-degenerate parts of the surface. These fluxes are then extended to the entire structure, resulting in a full description. However, this approach only works for symmetric geometries and fails once even small irregularities, such as surface roughness, break the symmetry, which is unavoidable in most practical simulations.

**Figure 10:**
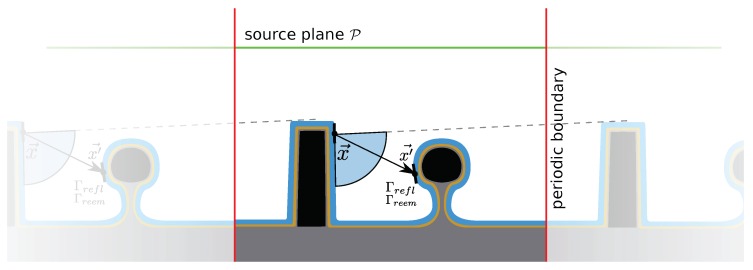
Calculation of reflected or reemitted fluxes using a bottom up technique with periodic simulation boundaries. The black arrow indicates the direction used to find the reflected and re-emitted flux incident on $\vec{x}$ from a particle source at $\vec{x^{\prime }}$. The source distributions $\Gamma_{refl}$ and $\Gamma_{reem}$ define the flux emitted towards $\vec{x}$. The total indirect flux at point $\vec{x}$ is found by summing the flux from all visible surface points, highlighted by the blue arc.

Therefore, iterative approaches are commonly used, which first calculate the direct flux $F_{0}$ and then the fluxes to be reflected $F_{refl}$ or re-emitted $F_{reem}$ from each discretized surface element. All of the discretized surface elements can then be described as a particle source with distributions $\Gamma_{refl}$ and $\Gamma_{reem}$, which is shown schematically in Figure 10. These source distributions also include the description of the reflected and re-emitted particles in terms of angular dependence and other surface properties. The contributions from a particle source at $\vec{x^{\prime }}$ visible to $\vec{x}$ can then be found using $\Gamma_{refl}$ and $\Gamma_{reem}$. All of these contributions are summed to give the total incident flux, similar to the direct flux calculation, which leads to an expression for the total reflected and re-emitted flux incident on $\vec{x}$. The total flux after the first iteration $F_{1}$ is thus:

(5) $$F_{1}\left(\vec{x}\right)=\sum_{\vec{x^{\prime }}}Y\left(\vec{x^{\prime }},\vec{x}\right)\left[\Gamma_{refl}\left(\vec{x^{\prime }},\vec{x},F_{refl},E\right)+\Gamma_{reem}\left(\vec{x^{\prime }},\vec{x},F_{reem},E\right)\right].$$

This process can be repeated *n* times, until all particles have been adsorbed or a satisfying accuracy has been reached. The necessary number of iterations depends strongly on the properties of $\Gamma_{refl}$ and $\Gamma_{reem}$ as they dictate how much of the incoming flux is reflected or re-emitted again, respectively. The number of necessary iterations can also be set using a minimum change in arrived fluxes, which should be achieved at each iteration. If the fluxes change less than this margin, no further iterations are needed. The final result for the flux incident at $\vec{x}$ is given by

(6) $$F\left(\vec{x}\right)=\sum_{0}^{n}F_{n}\left(\vec{x}\right).\;$$

After each step, only the flux to be used in the subsequent iteration is saved. Therefore, specular reflections cannot be modeled accurately using the bottom-up method, but only by assigning an average incoming and thus outgoing direction [46]. Therefore, energetic ion reflections, which are mostly specular, cannot be modeled accurately using this method, as they are approximated by a common reflection distribution, $\Gamma_{refl}$.

### 2.4. Chemical Modeling

Similarly to the transport of molecular entities, discussed in Section 2.2, it is not feasible to simulate all chemical reactions taking place inside a reactor. In order to simplify the modeling complexity, usually only surface reactions are considered [68], while reactions in the gas phase are assumed to reach a steady state due to the short reaction times compared to the time it takes to traverse the reactor. In order to find the effective particle flow, captured by the source flux distribution $\Gamma_{src}$, it is essential for reactor-scale simulations to identify dominant reactions in the gas phase and properly approximate the particle flow to the surface. This flow can either be found experimentally or simulated for specific reactor geometries [69], which can indeed be challenging for complex processes. Due to the large number of different chemical elements used for each etching step during gate stack patterning and the high temperatures in the reactor, simulating the expected source flux distribution is cumbersome and time-consuming. Even if dominant reactions can be identified, simulating them in the energetic environment of a plasma reactor can present a challenge. Therefore, experimental data of these processes are vital for accurate simulations.

Simulating surface reactions can still be highly challenging as volatile atoms and molecules are usually involved in etch processes, creating a wide variety of possible end products. Especially modern plasma etch processes pose a challenge as many different chemical species are present, leading to countless reactions and thus great computational effort required to find reasonable results. Since modern gate stack etch sequences consist of a combination of such processes, which can influence each other strongly, physically meaningful models must consider highly complex chemical phenomena. Even if all possible reactions could be modeled efficiently, the wide variety of reactor and surface geometries, as well as high sensitivity to minor changes in chemical composition of reactants, makes it impractical to gather meaningful data from experiments in order to test computational models. Therefore, semi-empirical models are still the most robust options in simulating modern fabrication processes.

Each of the different physical processes involved are usually described by coefficients in a general surface rate model, which can then be used to find the overall surface normal velocity $v(\vec{x})$ [70]. These coefficients must be fitted to a particular technology by comparing them to fabricated structures [71]. For an arbitrary plasma etch simulation, all possible physical processes have to be taken into account, including chemical etching, ion-enhanced etching, sputtering, and deposition. Depending on the actual properties of each of these processes, different coefficients are used to describe them. To simplify the modeling and to allow for a description in discrete time steps, necessary for process simulations, the effect of every physical process on the surface can be computed by considering the relative concentrations of materials on the surface, found using flux calculations described in Section 2.2.

**Figure 11:**
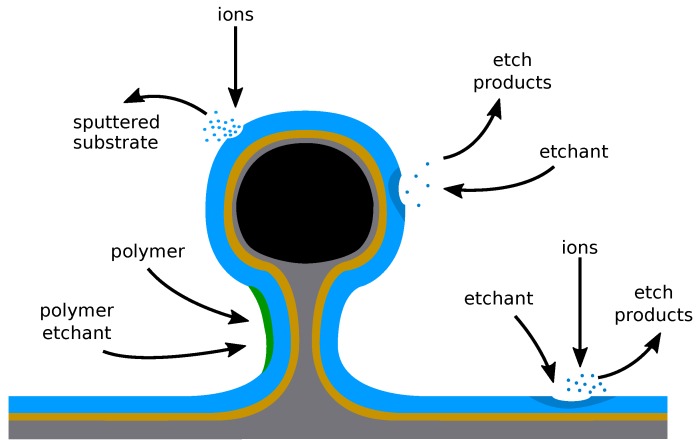
Four physical processes, which are considered when describing a modern plasma etch process used for gate stack pattering. Passivating species form polymer layers on sidewalls, as there are fewer energetic ion impacts to remove them from these surfaces. Ion sputtering removes material from the substrate by physical sputtering without the involvement of a chemical etchant. Purely chemical etching removes material by forming volatile etch products, which then desorp from the surface. Ion-enhanced etching speeds up this process by breaking existing bonds, enhancing the formation of volatile etch products.

For further simplification, the chemicals involved are grouped into a smaller number of types, representing their effect on the surface [72]. The rates used for simulation usually describe neutral etchant particles, passivating particles, passivation etchant particles, and ions, as illustrated in Figure 11. Re-emitted etch products and sputtered material are usually included during ray tracing and thus affect surface fluxes directly, without the need for any further considerations.

The rates of particle types impinging on the surface can be summed to give coverages of different particle types, $\phi_{x}$, where *x* is a particle type. Therefore, coverages of etchant $\phi_{e}$, polymer $\phi_{p}$, polymer etchant $\phi_{pe}$, and ions $\phi_{i}$ describe the amount of material covering this surface. Stochastically, this can also be seen as the probability of a given particle being at that location on the surface. Although ions would not deposit and cover the surface, $\phi_{i}$ is used to capture the number of ions which impinged on the surface, described as a coverage here for simplicity. The coverages can then be used in different models to find the surface normal velocity $v(\vec{x})$ described in Section 2.1, hence describing etching or deposition. Assuming steady-state conditions for the different surface coverages, a system of linear equations describing all physical deposition and etching processes is set up [73]:

(7) $$\frac{d\phi_{e}}{dt}=J_{e}S_{e}\left(1-\phi_{e}-\phi_{p}\right)-k_{ie}J_{i}Y_{ie}\phi_{e}-k_{ev}J_{ev}\phi_{e}\approx 0,$$

(8) $$\frac{d\phi_{p}}{dt}=J_{p}S_{p}-J_{i}Y_{p}\phi_{p}\phi_{pe}-\Delta_{p}\approx 0,$$

(9) $$\frac{d\phi_{pe}}{dt}=J_{e}S_{pe}\left(1-\phi_{pe}\right)-J_{i}Y_{p}\phi_{pe}\approx 0.$$

Each term in Euqaitons (7)–(9) describes a physical process, which changes the surface, and includes the necessary coefficients, where $J_{x}$ denotes the respective arriving fluxes on the surface element, $S_{x}$ the respective sticking probabilities, $Y_{x}$ the yields (e.g., etching or sputtering yield), and $k_{x}$ are the stoichiometric factors, which describe how much of one material, compared to its reactant, is needed to form the reaction product. Sticking probabilities and coverages are bound to the range [0,1], where 1 stands for a fully covered surface, or fully balanced polymer by etchant in the case of $\phi_{pe}$, since this coverage is normalized to $\phi_{p}$. $\Delta_{p}$ describes the amount of material needed to advance the surface through deposition. The first terms in Euqaitons (7)–(9) describe the incoming flux adsorbed onto the surface, where the sticking coefficient describes the probability of adsorption. The second terms in the above equations are proportional to the ion flux and describe the loss of particles through ion enhanced etching, which may remove all types of particles from the surface. However, the removal of material through evaporation or chemical etching is only considered for the etchant species, since it reacts to form compounds, which, by definition, have a much lower binding energy to the surface than other materials present on the surface [74].

The change in surface coverages must include all relevant mechanisms which add or remove particle types from the surface. Since mass cannot be lost, the number of particles arriving and departing from the surface must balance [75]. Therefore, the surface coverages reach a steady state with respect to etching and deposition time scales almost instantaneously.

Considering the polymer coverage, as shown in Equation (8), the first term is directly proportional to the deposition rate $DR_{p}$ of the polymer,

(10) $$DR_{p}=\frac{1}{\rho_{p}}\left(J_{p}S_{p}\right),\;$$

while the second term is proportional to the etch rate $ER_{p}$ of the polymer,

(11) $$ER_{p}=\frac{1}{\rho_{p}}\left(J_{i}Y_{p}\phi_{p}\phi_{pe}\right).\;$$

If the etch and deposition rates balance perfectly, no polymer will be deposited since $\Delta_{p}=0$ and there is no material left for deposition. If more particles impinge than are removed through ion-enhanced etching, the additional material will deposit onto the surface, advancing the surface at $\vec{x}$ by a velocity

(12) $$v\left(\vec{x}\right)=\frac{\Delta_{p}}{\rho_{p}}=\frac{1}{\rho_{p}}\left(J_{i}Y_{p}\phi_{pe}-J_{p}S_{p}\right).\;$$

In Equation (12), $\rho_{p}$ represents the density of the polymer, and $\phi_{p}$ is ignored as it must be unity if the entire surface is covered by the polymer. Since $\phi_{pe}$ can be found easily from Equation (9), the surface normal velocity field $v(\vec{x})$ necessary to advance a surface, as described in Section 2.1.2, can be found straightforwardly.

The same field can be constructed using Equation (12), if etching dominates, provided the underlying material is the polymer. If all the polymer has been removed and the substrate is etched, different effects must be taken into account, which depend on the specific etch chemistries used. Assuming that the substrate can be removed by chemical etching, ion-enhanced etching, and physical ion sputtering, as is the case for many plasma chemistries, the normal velocity of the surface at $\vec{x}$ is expressed as:

(13) $$v\left(\vec{x}\right)=\frac{1}{\rho_{sub}}\left[\underset{\mathrm{chemical}\;\mathrm{etching}}{\underbrace{J_{ev}\phi_{e}}}+\underset{\mathrm{ion}\text{-}\mathrm{enhanced}\;\mathrm{etching}}{\underbrace{J_{i}Y_{ie}\phi_{e}}}+\underset{\mathrm{ion}\;\mathrm{sputtering}}{\underbrace{J_{i}Y_{s}\left(1-\phi_{e}\right)}}\right].$$

The first two terms in Equation (13) describe loss mechanisms, which also remove etchant species from the surface coverage and therefore also appear in Equation (7), where the amount of etchant needed to remove a unit of the substrate is captured in the stoichiometric factors, $k_{x}$. The etchant coverage, $\phi_{e}$, depends on the other coverages in this case, as all of them are active and can be found by solving Equations (7)–(9). Therefore, surface normal speeds for polymer deposition and etching, Equation (12), as well as substrate etching, Equation (13), can be found using only the incoming particle fluxes, $J_{i}$, $J_{e}$, and $J_{p}$.

Additionally, each etching mechanism can be described more accurately by considering certain dependencies, such as ion-enhanced etching or ion sputtering, which depend strongly on the energy and incoming angle of ions [76]. The choice of coefficients for each process is the most crucial step and usually requires data from experiments, other reactor-scale or ab initio simulations, or a combination of both. A model encapsulating a large number of etch mechanisms could, in theory, describe numerous different processes. The choice of sticking probabilities, etch yields, and stoichiometric factors constitutes the only differentiating property for a variety of process models. They are therefore fitting parameters, which have a basis in the physical and chemical surface reactions taking place. Model calibration is therefore one of the most important and time-consuming parts in process simulation.

Any physical effect modifying the number of molecular entities arriving on the surface could be included, adding more coefficients and thus increasing the complexity, although simple models are frequently sufficient, even when describing complex processes [77]. It is therefore crucial to identify the dominant physical processes for each chemistry in order to make sure they are adequately represented in the model, as some effects might dominate the behavior of one chemistry, while they can be neglected in others. The model described above can be considered as an illustration of how such a model might be set up and does not describe all potentially relevant physical effects exhaustively. At the same time, some chemistries might already be described well in a simpler model, such as pure chlorine etching, where the effect of ions might be negligible compared to the chemical etching properties. The dominant mechanisms during each patterning step of gate stacks, the required modeling techniques, and their dependence on the basic concepts introduced earlier are discussed in the next section.

## 3. Simulation Software

Several proprietary and open-source simulation frameworks with the capability to describe the complex processes occurring in gate stack patterning, are readily available. Some well-known frameworks and the numerical methods on which they are based, are discussed here briefly:

**Sentaurus Topography** [78] is a commercial simulator developed by *Synopsys* (Mountain View, CA, USA), which uses level set based surface descriptions for topography changes, cell based representations for chemical surface reactions, and provides Monte Carlo methods for particle transport [79]. Dunn et al. [80] successfully used this tool to simulate the fabrication of FinFET structures of the 7 nm node. The Florida Object Oriented Process Simulator (FLOOPS) [81] provides similar capabilities. It was incorporated into Sentaurus, but a version is also available as an open-source project.
**Victory Process** [82] is a proprietary process simulator distributed by *Silvaco* (Santa Clara, CA, USA). It allows level set surface descriptions, as well as explicit surfaces to be used. Nanda et al. [83] were able to simulate the fabrication of strained FinFETs using this framework. Victory Cell [84] is a related tool, which uses cell based and explicit surface representations in order to improve the description of ion implantation and diffusion. It was used by Maiti et al. [85] to simulate the fabrication of stressed FinFETs.
**ViennaTS** [86] is an open-source feature scale process simulation tool developed at the *Institute for Microelectronics*, *TU Wien*. Surfaces are represented using level sets and top-down, as well as bottom-up methods, are implemented. The software provides predefined etch and deposition models, including several for the simulation of advanced node etching processes [77].
**The Monte Carlo Feature Profile Model (MCFPM)** [87] is one of several software components developed at the *Computational Plasma Science and Engineering Group, University of Michigan*. Cell based methods are used to describe different materials and top-down approaches are used to describe particle transport. Combined with other software components, it was used by Huard et al. [88] to simulate the fabrication of advanced-node FinFETs.

Phietch [89] and K-Speed [90] are also widely used simulation frameworks, while SEMulator3D [91] provides a framework for process emulation. University and open source tools, such as ViennaTS and MCFPM, usually provide the underlying methods, algorithms and implemented models of the framework. Commercial tools usually do not disclose these. Therefore, the methods discussed in this review are primarily based on studies performed with open academic tools.

## 4. Plasma Chemistries for Gate Stack Etching

The etching sequence of a gate stack used for advanced technology nodes of 14 nm and below consists of several highly different processes with unique properties and etch mechanisms due to the different materials used in the gate stacks [92]. It includes highly anisotropic dry etch processes, as well as highly selective or isotropic ones, depending on the different materials included in the gate stack [93]. Wet etch processes have generally fallen out of favor in advanced node gate stack patterning due to additional cleaning steps required to remove residues left on the wafer after wet etching. Furthermore, their isotropic etch properties are not ideal for the high vertical etching accuracy needed for modern three-dimensional structures [94]. Therefore, widely adopted process flows in industry today rely on dry plasma etch processes [95].

**Figure 12:**
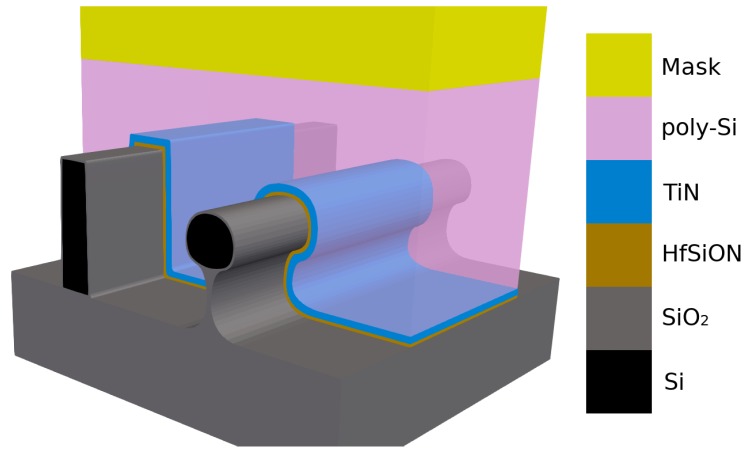
Schematic depiction of multi-layered geometries of modern three-dimensional gate structures after gate etching. A trigate (left) will have three sides of the Si-channel accessible to the gate, while an Ω-gate (right) comes close to an all-around gate structure.

Even though two processes may have similar etch properties, the underlying mechanics might differ completely, leading to diverse effects in complex geometries or chemically different environments. In the following, the applicability and reliability of the earlier introduced concepts to these real processes will be discussed in reference to advanced node metal gate stacks. These metal gate stack geometries, shown in Figure 12, usually consist of a thin layer of high-k dielectric, such as hafnium dioxide, a contact metal, such as titanium nitride, and poly-Si [94,96]. These layers cover the conducting silicon channel, on top of an insulating silicon dioxide substrate. In order to achieve better switching characteristics, the contact area between the gate and the conducting channel should be maximized An established approach to achieving these high contact areas, while reducing the footprint on the wafer, is using three-dimensional structures, such as the ones shown in Figure 12: a trigate, where the channel is as thin and high as possible [97], and an Ω-gate [98], where the channel is almost completely surrounded by the gate. The variety of the incorporated materials leads to the need for a sophisticated and carefully tuned etch sequence, removing each layer without damaging masked regions or the layers below. Gate all-around (GAA) structures and stacked nanowire gates, which achieve a gate contact around the full circumference of the channel, are researched heavily as a promising improvement on the finFET and omega gate structures [99]. Many different approaches exist, some incorporating a variety of new materials. Therefore, the specific materials and the fabrication techniques, which gate all-around structures might incorporate, are not discussed here. However, many of the current etch techniques will likely also be applicable to the fabrication of GAA structures [100].

### 4.1. Silicon Etching

As silicon is the most important material in semiconductor fabrication, many different etching chemistries have been investigated in the past several decades [101,102]. In the following, the three most common chemistries for directional dry etching of silicon are described and their etching mechanics as well as possible use in a gate stack patterning sequence is discussed.

#### 4.1.1. CF Type Chemistries

**Figure 13:**
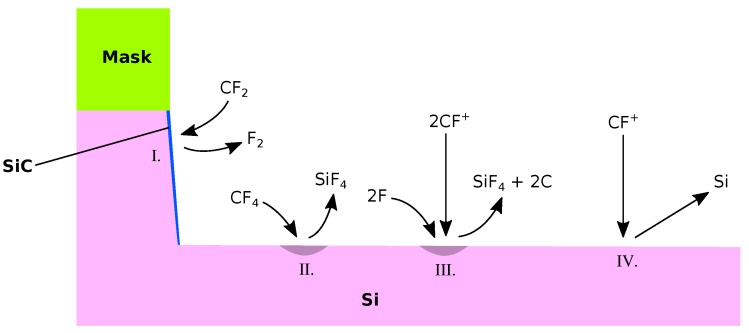
Active etching and deposition mechanics in CF type chemistries used to etch poly-Si: I. chemical deposition of carbon forming an SiC passivation layer, II. chemical etching, III. ion-enhanced etching, and IV. ion sputtering through high energy ions.

Fluorocarbon (CF) chemistries have been used to etch Si and SiO_2_ for decades, due to the ability of fine tuning of different materials’ etch rates, thereby improving selectivity [103]. The use of several additive gases allows for the etch processes characteristics to change drastically [104], which enables the adjustment to better fit a variety of substrates and geometries. In this manner, a high etch selectivity can be reached for certain materials, in addition to the highly anisotropic properties of plasma etch processes [105]. Fluorocarbon plasmas can etch chemically, via ion-enhanced etching or physical sputtering, which is shown in Figure 13. Silicon is removed chemically by reacting with fluoride leading to the etch products evaporating back to the gas phase [106]. The rate of chemical etching depends on the temperature and is reduced by carbon atoms present on the surface. Ion-enhanced etching proceeds through the bombardment of radical CF^+^ ions reacting with the substrate, which are either sputtered from the surface or evaporate due to their now smaller binding energy to the surface [107]. Physical sputtering appears only above a threshold ion energy, which is related to the binding energy of the substrate. Deposition takes place through the polymerization of neutrals, covering the surface by forming SiC bonds, or through direct ion deposition, where energetic ions are directly absorbed into the substrate [108]. Thus, most active mechanisms in this chemistry can be described by the illustrative model given in Section 2.4. Only direct ion implantation cannot be included when using level sets, as there is no volume information. However, it can be included in a model when using explicit volume definitions or voxel elements.

#### 4.1.2. SF Type Chemistries

**Figure 14:**
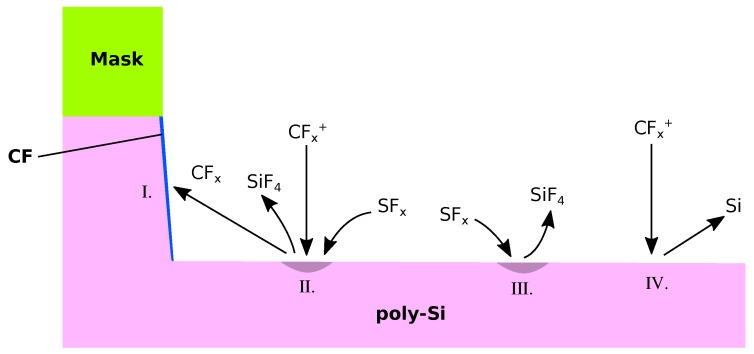
Sulfur with Fluoride (SF) type etching and deposition mechanics with additional CH_2_F_2_ feed gas. I. Line of sight deposition of a CF passivation layer; II. ion-enhanced etching; III. chemical etching; and IV. physical ion sputtering.

Sulfur Hexafluoride (SF_6_) is a good alternative to CF chemistries because of their high etch rates and due to the fine control over the etch properties using additional gases fed into the reactor [109]. Pure SF_6_ chemistries etch isotropically, while the addition of oxygen (O) forms a thin silicon oxide passivation layer, which inhibits lateral etching [110]. Oxygen also binds sulfur, prohibiting the recombination with fluoride, more of which is then available for etching, resulting in higher vertical etch rates [111]. However, if the O concentration is too high, it competes for surface adsorption with F, reducing the etch rate [112]. Etching proceeds only on lateral surfaces due to the ion bombardment preventing the buildup of a passivation layer. Fluoride atoms can then attach to silicon atoms on the surface, forming SiF_4_, which is then removed chemically or through ion-enhanced processes [113]. If only oxygen is used as an additional gas, the physical processes can be described straightforwardly. However, the introduction of additional gases can create more complex properties. For example, the addition of hydrogen bromide (HBr) and oxygen results in better sidewall passivation and less lateral etching, due to the formation of a SiO_x_Br_y_ passivation layer, which reflects high energy ions, further enhancing the vertical etch rate [114]. This additional interplay of different chemical compounds has to be considered carefully when developing a model and cannot be represented with such a simple description as used in Section 2.4. Passivating species other than oxygen, such as CH_2_F_2_, have been used successfully to etch silicon [115]. The formation of sidewall passivation in such a chemistry is not due to the deposition from the gas phase, but rather due to sputtering of CF etch products from vertical etching and line of sight deposition [116], shown in Figure 14. Modeling this process requires an additional ray tracing step which could be realized by launching rays from a surface element, if a certain combination of etchant and ion coverage is reached. The additional ray tracing required decreases computational efficiency, but is indispensable in order to model the shadowing effects expected in complex geometries. Approximating the build-up of the passivating layer with deposition from the gas phase is only sufficient in simple geometries [77]. Hence, the modeling of most modern gate geometries require additional ray tracing for an accurate description of the deposition mechanism.

#### 4.1.3. HBr Type Chemistries

**Figure 15:**
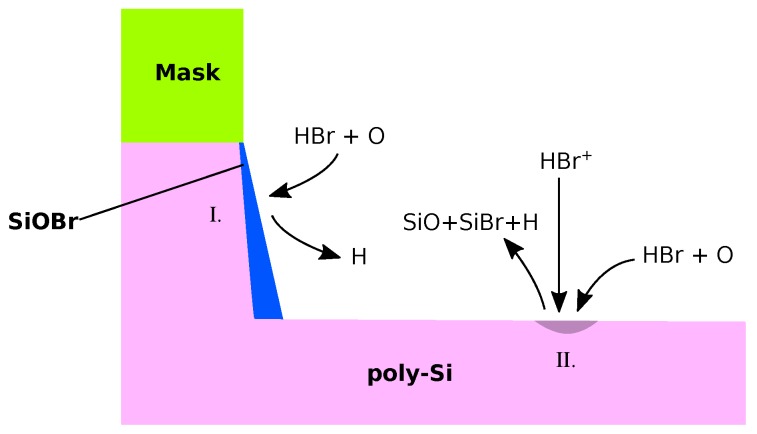
Dominant etch mechanics during silicon etching using hydrogen bromide. I. Sidewall passivation proceeds though chemical deposition from the gas phase, while II. vertical etching is dominated by ion-enhanced etching.

When etching a layer of the gate stack, care must be taken in order not to damage the layer below [117]. Poly-Si etching in a metal gate stack is therefore often concluded with a more selective HBr chemistry, despite its lower etch rate [118]. Even before metal gate stack designs, HBr/O_2_ chemistries were used due to the high selectivity against the gate oxide, used as a dielectric [119,120]. This chemistry is quite simple as ion-enhanced etching typically dominates, meaning other etching mechanisms can be ignored, while deposition proceeds mainly chemically [114]. As shown in Figure 15, thick SiO_x_Br_y_ layers form on sidewalls and the gate oxide, protecting it from energetic ions. Over the course of the etch process, bromine is removed from the passivation layer and replaced by oxygen, resulting in a denser silicon oxide layer on top of the passivation layers formed earlier in the process compared to an amorphous, bromine rich layer at the side walls formed later [121]. Modeling this desorption of bromine from the surface is not easily achievable, as the densening depends on the fraction of bromine in the passivation layer, which is a volume property, making direct simulation impractical when using implicit surfaces. Since a cleaning step often follows, it might be sufficient to only model the final oxide layer using direct deposition [122].

### 4.2. TiN Etching

Titanium has been used in microelectronics as a mask, as well as an interconnect layer, for decades. Therefore, appropriate etch chemistries were investigated long before its introduction in metal gate stacks [123,124]. Due to the high temperatures required for higher etch rates in fluorinated plasmas, chlorine or bromine based chemistries are applicable more universally [125]. TiN is removed predominantly via ion-enhanced etching [126], forming unstable TiCl/TiBr etch products, which quickly react to other, more stable compounds such as TiO [127] in the presence of oxygen. Fast oxidation of the Ti surface can form an etch stop layer due to the much lower etch rate of TiO_2_ [128]. It is therefore important to reduce the amount of oxygen when etching titanium compounds [129], despite the increased etch rate of passivation layers. However, small amounts of oxygen result in a higher fraction of ionized etchant in the gas phase, increasing the etch rates and selectivity below a concentration of 1%. Bromine etches titanium significantly slower than chlorine due to its lower volatility, while HBr can be added to Cl chemistries to achieve better control over certain etch properties [126]. Different additives, such as boron (B) can also be included, which forms non-volatile BO_x_N_y_ polymers chemically, protecting TiN sidewalls and further reducing lateral etching [130]. Due to the relative simplicity of the etching and deposition mechanics, simulation of TiN etching is possible with the model described earlier. However, care must be taken when experimenting with feed gases other than the ones described, as they might change the underlying mechanics drastically. The models would need to be adjusted accordingly and are not predictable across different feed gases, due to the potential changes in the underlying mechanics.

### 4.3. HfO_2_ Etching

Since the dielectric layer is usually very thin, high etch rates are not as important as high selectivity to the silicon substrate and the other materials in the gate stack. Fluorine based chemistries have been shown to reach satisfactory selectivity and etch rates [131], although CF based chemistries form thick fluorocarbon layers, requiring an additional cleaning step after etching, which is not the case for SF chemistries [132]. The thickness of these layers could be reduced by adding hydrogen, which removes carbon from the surface via loosely bound hydrocarbon etch products [133], although even these fewer fluorocarbon residues on the substrate still present a problem [134].

**Figure 16:**
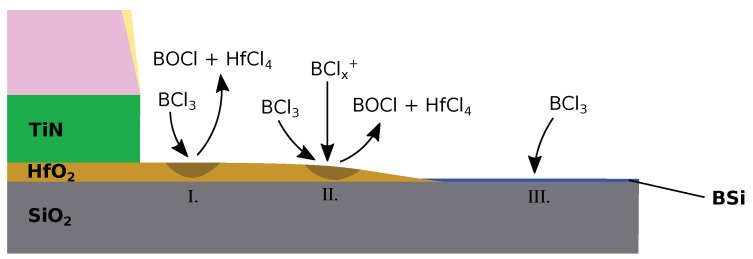
Etch mechanisms leading to infinite etch selectivity of HfO_2_ over SiO_2_ through I. chemical and II. ion-enhanced etching of HfO_2_, with III. additional chemical deposition of BSi on the SiO_2_ substrate.

Such problems are not encountered in HBr or Cl based chemistries, due to the surface bonding energies of their etch products [135]. However, due to the small thickness of the HfO_2_ layer, a high dielectric to substrate selectivity is necessary in order to meet the requirements for transistors, while these chemistries only offer selectivities of around 15, even when carbon is added as an inhibitor [136]. BCl was initially added to pure Cl chemistries to achieve higher selectivities [137], while pure BCl_3_ chemistries were soon chosen due to their near-infinite selectivity to the substrate. This is due to the favored reaction of the present boron with oxygen and silicon over hafnium, forming SiB compounds on the surface of the substrate, but no boron layers on Hf, which is shown in Figure 16. Therefore, the surface of the dielectric is free for chlorine to attach and form volatile etch products, removed via ion-enhanced etching, while the silicon and silicon dioxide surfaces are covered in an SiB layer, prohibiting the further adsorption of etching species. This layer is only removed via high-energy ions, so near-infinite etch selectivities towards Si and SiO_2_ can be observed at low bias powers [138]. Although BCl_3_ etching is mainly enabled by ion-enhanced etching, the strong dependence on ion energy with respect to selectivity, requires careful fitting of model parameters to the observed rates.

### 4.4. Full Etch Sequence

**Figure 17:**
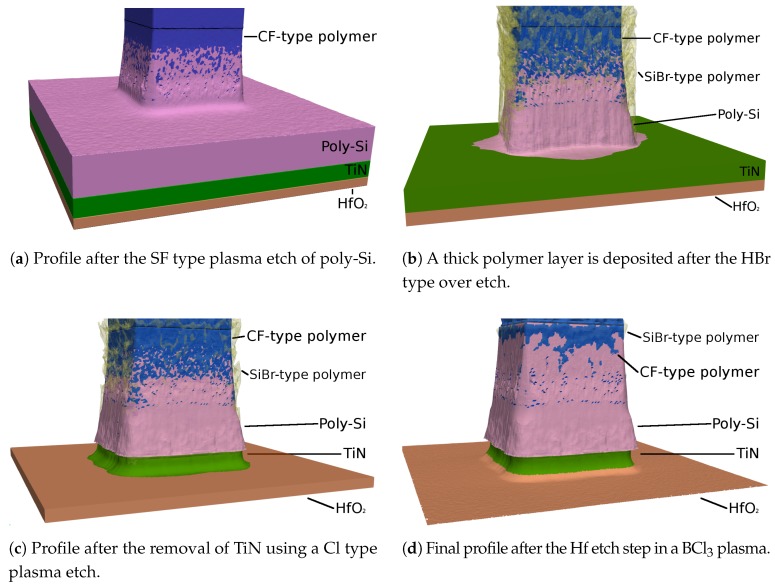
Simulated gate stack geometry after each etch step, showing the influence of passivation layers on subsequent etch steps. The poly-Si sidewalls remain protected during the more isotropic later etch steps, which leads to the under-etch in the TiN and Hf layers.

Since several of the etch steps described above involve the deposition of passivation layers to protect side walls, the subsequent etch steps can be affected by these residues. Therefore, simulating the combination of all these processes can provide an insight into the full process, including the effects of each etch step on the subsequent ones. As we [77] have documented using the simulator ViennaTS, the final profile of a 14 nm fully depleted silicon-on-insulator (FDSOI) gate stack patterning process is indeed strongly influenced by earlier etch steps, which involve similar chemistries to the ones described above. This influence is shown in Figure 17, which highlights the role of deposited passivation layers for subsequent etch steps in protecting the otherwise exposed poly-Si sidewalls. While the poly-Si is protected by the earlier deposited SiBr and CF layers, the HfO_2_ etch step creates an under-etch in the TiN layer since it is not protected. After the final etch step, only remnants of the protecting layers remain, which are removed in a subsequent cleaning step.

Figure 17 also shows why the ability to represent very thin layers is important when modeling gate stacks, since several materials with highly different etch properties must be considered in these simulations. Even the thin layer of CF, deposited in the first etch step, can protect the poly-Si in the last step through its much lower etch rates. Thus, the correct representation of thin layers by the choice of adequate surface description techniques is crucial in this application.

**Figure 18:**
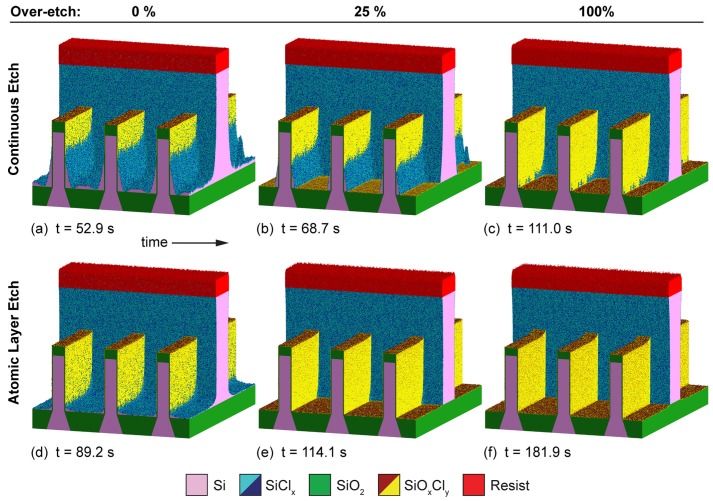
Profiles resulting from continuous Cl etching (**a**–**c**) and self-limited atomic layer etching using the same chemistry (**d**–**f**); used with publisher’s permission from [88].

Due to the small layer thickness, which might be comparable to a few atomic sizes, the thin layers do not have sharp material boundaries, but rather consist of a mix of materials. Therefore, the simulated thin layers should be seen as a rough guide to the amount of material present at the interface, rather than abrupt changes in composition. In order to circumvent these sharp boundary representations, rectilinear explicit meshes, as described in Section 2.1.3, can be used to represent single blocks of compounds at the interface. Huard et al. [88] successfully used the MCFPM based on this approach, as described in Section 3, to simulate the gate etching of finFET structures. They compared the profiles created by continuous etching and self-limited atomic layer etching of silicon using ion-enhanced chlorine chemistries. Diffusion and ion-implantation are taken into account, enabled by the cell based surface description. The difference in the resulting layers to level set based simulations can be seen clearly in Figure 18, which shows the thin passivation layers as a mixture of different materials, rather than a single strictly defined layer. However, due to the fixed size of each cell, sharp material boundaries can only be avoided if the cell size is comparable to atomic sizes. Modeling at these scales also enables the inclusion of additional interactions between cells, creating a better physical description. However, the only way to truly simulate the physical behavior is atomistic modeling, which takes into account single atoms and bonds.

## 5. Conclusions

Modern gate stack patterning processes require sophisticated simulation techniques, starting from the fundamental computational methods used, due to the numerous new materials introduced into the gate stack. In order for complex deformations, as expected in modern dry etch processes, to be represented efficiently, several surface representations are considered for different purposes. While moving surfaces are described more robustly using implicit surface representations, such as level sets or cell based methods, ray tracing techniques used for flux calculations are better suited for explicit surface representations. However, the level set method is still the most widely used technique to describe interfaces in process simulations, due to its robustness during complex deformations. Furthermore, approximate explicit surface descriptions using disks or spheres eliminate the need for computationally expensive conversions to triangulated surfaces for rate calculations with ray tracing techniques.

Robust surface descriptions and physically meaningful rate calculation methods made recent advances in the modeling of gate stack etching processes possible. The correct representation of thin layers, often formed in such processes, contribute to the robust description of sequential etching steps, while ray tracing techniques allow for accurate and physically more meaningful modeling of the transport of molecular entities to the etched surface.

Furthermore, advances in the identification of the dominant etching and deposition mechanics allow for more physical representations of complex etch chemistries in reactor- and feature-scale simulations. The main factors defining the properties of different dry etch techniques and chemistries can thus be identified and incorporated into universal models, allowing for the physically meaningful description of many modern etch processes. Even if the dominant mechanics are identified, some physical processes might not be described adequately in certain surface representations, such as ion-implantation using level sets. Due to the lack of volume information in level set surfaces, ion-implantation cannot be modeled straightforwardly. Explicit or cell based methods are more accurate in describing volume dependent processes, but require more sophisticated methods to advance the surface.

One of the biggest challenges faced today is the synthesis of predictive models over a wide range of reactor geometries, etch chemistries, and feature scale profiles. Due to the complexity of the chemical processes taking place inside the reactor, minor changes in process parameters can result in drastic changes in the etch profile. While process simulation already provides an understanding of the dominant chemical processes by comparison with experiments, the prediction of results requires highly sophisticated surface descriptions, as well as chemical models. Advances in computer performance and numerical models will enable atomistic approaches, where each atom or molecule is described during etch processes. This level of sophistication will likely enable the development of truly predictive models, reducing the need for expensive experiments in order to create advanced node structures.
